# Highly Selective Electrochemiluminescence Sensor Based on Molecularly Imprinted-quantum Dots for the Sensitive Detection of Cyfluthrin

**DOI:** 10.3390/s20030884

**Published:** 2020-02-07

**Authors:** Jinjin Xu, Rongrong Zhang, Chenxi Liu, Aili Sun, Jiong Chen, Zeming Zhang, Xizhi Shi

**Affiliations:** 1 Laboratory for Managing Biotic and Chemical Threats to the Quality and Safety of Agro-Products, Ningbo University, Ningbo 315211, China; jinjinxu827@163.com (J.X.); zhangrong19910510@163.com (R.Z.); 17815936495@163.com (C.L.);; 2 College of Food and Pharmaceutical Sciences, Ningbo University, Ningbo 315211, China; 3 School of Marine Sciences, Ningbo University, Ningbo 315211, China; sunaili@nbu.edu.cn

**Keywords:** molecularly imprinted polymers, quantum dot, cyfluthrin, electrochemiluminescence sensor, fish samples

## Abstract

A highly selective and sensitive molecularly imprinted electrochemiluminescence (MIECL) sensor was developed based on the multiwall carbon nanotube (MWCNT)-enhanced molecularly imprinted quantum dots (MIP-QDs) for the rapid determination of cyfluthrin (CYF). The MIP-QDs fabricated by surface grafting technique exhibited excellent selective recognition to CYF, resulting in a specific decrease of ECL signal at the MWCNT/MIP-QD modified electrode. Under optimal conditions, the MIECL signal was proportional to the logarithm of the CYF concentration in the range of 0.2 µg/L to 1.0 × 10^3^ µg/L with a determination coefficient of 0.9983. The detection limit of CYF was 0.05 µg/L, and good recoveries ranging from 86.0% to 98.6% were obtained in practical samples. The proposed MIECL sensor provides a novel, rapid, high sensitivity detection strategy for successfully analyzing CYF in fish and seawater samples.

## 1. Introduction

Cyfluthrin (CYF), a synthetic type II pyrethroid insecticide, was widely used in agricultural pest control; this insecticide could also enter aquatic ecosystems from agricultural areas via run-offs [1,2]. CYF residue was frequently detected in aquatic environments and organisms due to its widespread usage and high persistence [3,4]. Although CYF featured low mammalian toxicity, long-term exposure to this chemical caused a toxic effect on the respiratory, nervous, immune, and reproductive systems of human beings and nontarget organisms [5]. Due to these risks, various countries had stipulated their maximum CYF residues (0.05 mg/kg in food established by China; 0.1 mg/kg in eggplant by EU; 0.01 mg/kg in farmed fish by Brazil) and banned its usage in aquaculture [6,7,8]. Therefore, a rapid, efficient, and sensitive method for detecting CYF residues in food and environment samples must be developed.

**Scheme 1:**
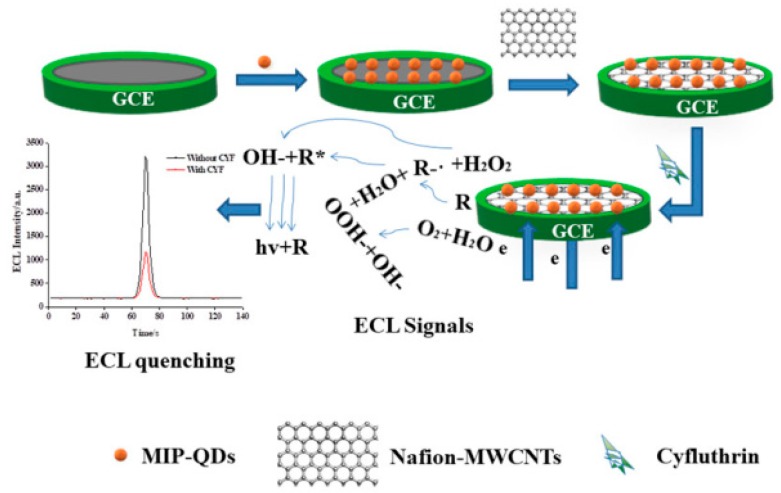
Schematic representation of MIECL sensor development.

At present, numerous confirmatory methods including gas chromatography tandem mass spectrometry (GC-MS/MS) and liquid chromatography tandem mass spectrometry (LC-MS/MS) have been successfully applied to CYF determination [9,10]. Although these methods have shown excellent sensitivity and selectivity, they also require expensive instruments and time-consuming, sophisticated sample pretreatments, causing difficulties in saving time for unexpected food safety incidents; hence, these methods have been deemed unsuitable for rapid field detection [11,12]. Recently, electrochemiluminescence (ECL), also called electrogenerated chemiluminescence, attracted considerable attention from researchers due to its simpler, higher sensitivity, more precise reaction kinetics, and better controllability and reproducibility compared with other electroanalytical detection techniques [13]. ECL is a particular process in which species are triggered at electrodes by high-energy electrochemical reactions to form excited states that emit light. Furthermore, the electrochemical reactions take place between the redox products of emitters and a co-reactant, generating an excited state which might decay and emit light [14,15]. ECL emission was initially generated through luminol and ruthenium (II) complexes; then, increasing attention was given to nanomaterials, such as quantum dots (QDs), carbon nanodots, metal organic gels (MOGs), and noble metal clusters [16,17]. Notably, QDs possess unique optical and electronic properties, such as high quantum efficiency, photobleaching resistance, and high electron transfer efficiency, and have been widely used in ECL systems [18,19]. To further improve the specific response capability and stability of QDs, in this work we combine molecularly imprinted polymers (MIPs) with ECL analysis based on QDs, showing high selectivity and good stability and controllability [20,21]. To date, QDs and MIPs have been individually fabricated and used as signal probes and recognition elements, respectively, resulting in a complex electrode preparation procedure and a remarkably negative impact on electrical conductivity [22,23]. Therefore, to simplify the electrode preparation procedure and improve electrical conductivity in the present study, we first proposed and fabricated MIP-QDs, which were synthesized by functionalizing cadmium selenide quantum dots (CdSe QDs) with molecular imprinting polymers as both the signal probe and specific recognition element of the ECL sensor for CYF determination. Furthermore, H_2_O_2_ was used as a co-reactant, and multiwall carbon nanotubes (MWCNTs) were utilized as reinforcements to provide excellent electrocatalytic activity and minimize surface fouling on the electrodes. Finally, the original MIECL sensor based on the MIP-QDs for CYF determination was developed and its application capability was fully evaluated. The results indicated that the fabricated MIECL sensor-based MIP-QDs demonstrated convenient, rapid, and accurate determination of trace CYF contaminants in fish and seawater samples. To the best of our knowledge, the use of a MIECL sensor for CYF determination based on MIP-QDs, H_2_O_2_, and MWCNTs has yet to be reported. Scheme 1 shows the principles of the developed method.

## 2. Materials and Methods

### 2.1. Materials and Reagents

CdSe QDs were purchased from BEIDA JUBANG Science & Technology Co., Ltd. (Beijing, China). The MWCNTs were supplied by XFNANO Materials Tech Co., Ltd. (Nanjing, China). Nafion (≥98.0%) was obtained from Sigma–Aldrich Trading Co., Ltd. (Shanghai, China). CYF, bifenthrin (BIF), deltamethrin (DEL), cypermethrin (CYP), and fenvalerate (FEN) were obtained from the Shanghai Pesticide Research Institute Co., Ltd. (Shanghai, China). 3-Aminopropyl-triethoxysilane (APTES), tetraethoxysilane (TEOS) and Triton X-100 were purchased from Sigma–Aldrich (Steinheim, Germany). H_2_O_2_ (AR, 30 wt. % in H_2_O) was obtained from Aladdin (Shanghai, China). All other reagents were of analytical grade and used with ultrapure water (resistivity ≥ 18.25 Ω). 

### 2.2. Instruments

Cyclic voltammograms (CVs) and the corresponding ECL experiments were carried out by using an ECL analysis system (Model MPI-E; Xi’an Remax Electronic Science & Technology Co., Ltd.; Xi’an, China). A conventional three-electrode system was used, consisting of a bare or modified glassy carbon electrode (GCE, 3 mm diameter), a saturated calomel electrode, and a platinum column electrode as the working, reference, and auxiliary electrodes, respectively. Electrochemical impedance spectroscopy (EIS) was investigated on an electrochemical workstation (CHI 660E; CH Instrument Company; Shanghai, China). The morphologies of the MIP-QDs and non-imprinted polymer QDs (NIP-QDs) were observed by transmission electron microscope (TEM; JEM-2100F; Hitachi Instrument; Hitachi, Japan).

### 2.3. Fabrication of MIP-QDs

The MIP-QDs were synthesized by following a modified micro-emulsion method, as previously reported in the literature [24]. Typically, 2.0 mL Triton-X 100 was dissolved in 8.0 mL cyclohexane and placed in a 50 mL round bottom flask. The mixture was magnetically stirred at 400 rpm for 15 min. Subsequently, 400.0 µL CdSe QDs (5.0 mg/mL), 50.0 µL TEOS and 100.0 µL ammonia were added in turn, and the solution was stirred for 2 h. Then, the mixtures of 110.0 µL CYF (25.0 mg/mL) and 22.8 µL APTES were prepared via magnetic stirring for 2 h under nitrogen atmosphere and then poured into a round-bottom flask. After stirring for 12 h, the MIP-QDs were synthesized and purified by adding 10.0 mL acetone to the reaction mixture, which was then centrifuged for 10 min at 9.0 ×10^3^ g. After discarding the supernatant, 6.0 mL ultrapure water was added and centrifuged at 9.0 × 10^3^ g for 20 min to remove the unreacted cross-linker and functional monomer. Finally, the template was removed with ethanol in acetonitrile (4:1, v/v) until no CYF could be detected by GC-MS/MS. The non-imprinted quantum dots materials (NIP-QDs) were simultaneously synthesized in the same process without the addition of template molecules.

### 2.4. Fabrication of the Nafion-MWCNTs/MIP-QDs/GCE

Prior to modification, the GCE was polished on a mirror surface with 0.05 µm alumina slurry, washed successively with absolute ethanol and ultrapure water, and ultrasonically shaken for 1 min to remove the polishing powder on the GCE surface. The treated bare GCE was then immersed in 100.0 mmol/L phosphate buffer solution (PBS, pH 7.0) and scanned at a rate of 100 mV/s over the potential range of −0.3 V to 0.4 V for five cycles. Then, the GCE was removed and ultrasonically washed in ethanol and ultrapure water to remove ions on the electrode surface. The GCE was coated with 10.0 µL of the resulting MIP-QDs (15.0 mg/L) suspension solution. After evaporation, 5.0 µL Nafion-MWCNTs (2.0 mg/L) solution was dropped onto the surface of the resulting MIP-QDs/GCE. Usually, Nafion is used not only as the binder to improve the adhesion of electrode sheet, but also as a mediator to facilitate the ionic transportation across the electrode [25]. After drying in air, the Nafion-MWCNTs/MIP-QDs/GCE was slightly washed with PBS (50.0 mmol/L) to remove the redundant modifier. As the control, the Nafion solution (0.5% mass fraction) and CdSe-QDs suspension were prepared to produce the Nafion/MIP-QDs/GCE and CdSe-QDs/GCE, respectively. Correspondingly, the NIPs with modified GCE were also fabricated under the same conditions. All modified electrodes were kept at 4 °C until use.

### 2.5. ECL Measurement

The MIECL sensor was used in a 10.0 mL homemade Teflon cell. The prepared sensor adsorbed the appropriate CYF concentration for 5 min, and CYF was then detected in 2.0 mL 100.0 mmol/L PBS (pH 9.0) solution containing 20.0 mmol/L H_2_O_2_. Cyclic voltammetry and ECL were used in the ECL emission process. The voltage, potential range, and scanning rate of the photomultiplier tube (PMT) were 800 V, −0.3–0.4 V, and 100 mV/s, respectively. CV scanning was performed in 5.0 mmol/L [Fe(CN)_6_]^3−/4−^ containing 0.1 mol/L KCl solution at the potential range of −0.3–0.6 V. EIS was conducted from 100 mHz to 100 kHz with an alternating current (AC) amplitude of 10 mV, which was used to characterize the different modified electrodes. The quenching efficiency of the MIECL sensor was defined as (Io − I)/Io, where Io and I represented the initial values of ECL intensity and the intensity of adding CYF, respectively.

### 2.6. Sample Preparation

The fish samples were purchased from a local supermarket in Ningbo. The fish samples (5.0 ± 0.01 g) were homogenized with a homogenizer at 8.0 × 10^3^ g. Subsequently, 15.0 mL acetonitrile and 1.5 g sodium chloride (NaCL) were added. After vortexing for 5 min, the samples were centrifuged at 4.0 × 10^3^ g for 10 min, and the procedure was repeated. Then, the supernatants were collected and mixed with 10.0 mL acetonitrile: n-hexane mixture (1:10, v/v) to further remove the fat and pigment contents. After vortexing for 5 min, the acetonitrile layer was separated by centrifugation at 4.0 × 10^3^ g for 10 min and blown to near dryness with nitrogen. Then the residue was diluted to 1.0 mL with acetonitrile for subsequent analysis.

Seawater samples were obtained from the offshores of Ningbo. The samples were filtered through a microporous nitrocellulose membrane (pore size 0.45 µm). The samples (5.00 ± 0.01 mL) were separately placed in 50 mL polypropylene centrifuge tubes, and the pH was adjusted to 9.0 for subsequent analysis.

## 3. Results and Discussion

### 3.1. Characterization of MIP-CdSe-QDs

**Figure 1:**
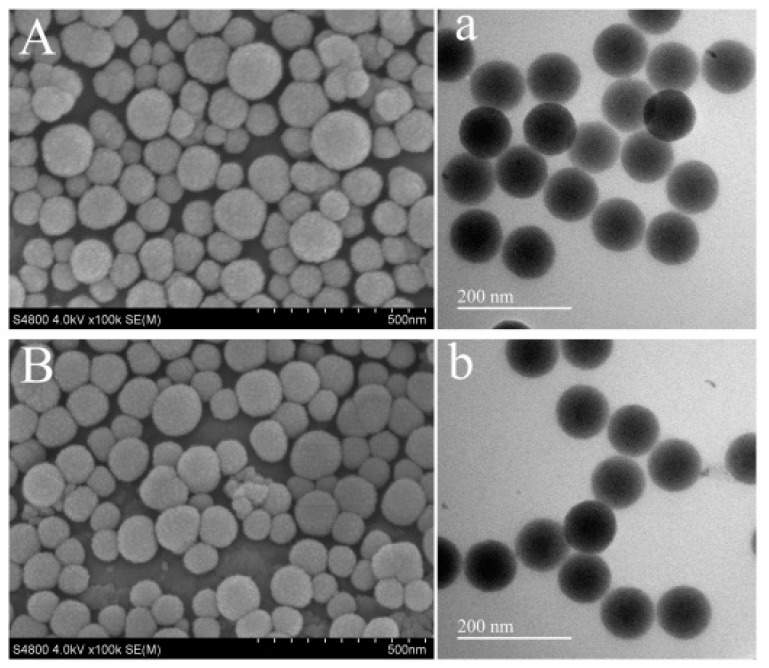
SEM images: (**A**) MIP-QDs and (**B**) NIP-QDs. TEM images: (**a**) MIP-QDs and (**b**) NIP-QDs.

**Figure 2:**
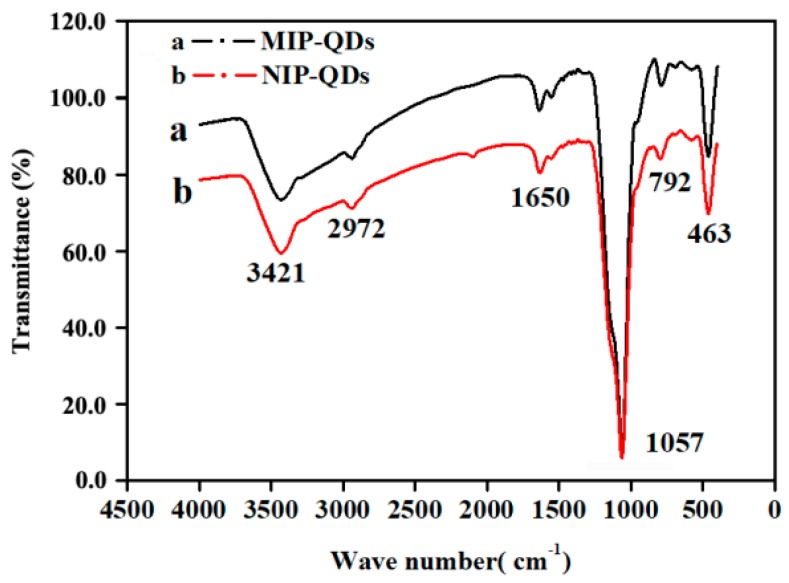
FT-IR spectra of MIP-QDs and NIP-QDs.

The corresponding sizes and morphologies of MIP-CdSe-QDs and NIP-CdSe-QDs were characterized by scanning electron microscopy (SEM) and TEM. As shown in Figure 1, the fabricated MIP-CdSe-QDs exhibited uniformly-sized microspheres with a diameter of approximately 100 nm. MIP-CdSe-QDs and NIP-CdSe-QDs presented rough surface morphology. No significant difference was found between the surface morphologies of MIP-CdSe-QDs and NIP-CdSe-QDs. In addition, to confirm the successful chemical modification on the QD surface, the Fourier transform infrared (FT-IR) spectroscopy of MIP-CdSe-QDs and NIP-CdSe-QDs were obtained and analyzed (Figure 2). The wide peak at 1057 cm^−1^ was assigned to the asymmetric stretching vibration of Si–O–Si. Other bands observed at approximately 792 and 463 cm^−1^ revealed Si–O vibrations. The peaks at approximately 2972, 3421, and 1650 cm^−1^ were consistent with the C–H and N–H stretching vibrations, indicating that the QDs were successfully embedded with a silica layer, which was formed by the sol-gel hydrolysis and condensation reaction of APTES and TEOS, and that the -NH_2_ groups of APTES were grafted onto the surface of the QDs [26]. Meanwhile, no significant differences were observed between the FT-IR spectra patterns between MIP-CdSe-QDs and NIP-CdSe-QDs.

### 3.2. ECL Behavior and Mechanism of the MIECL Sensor

**Figure 3:**
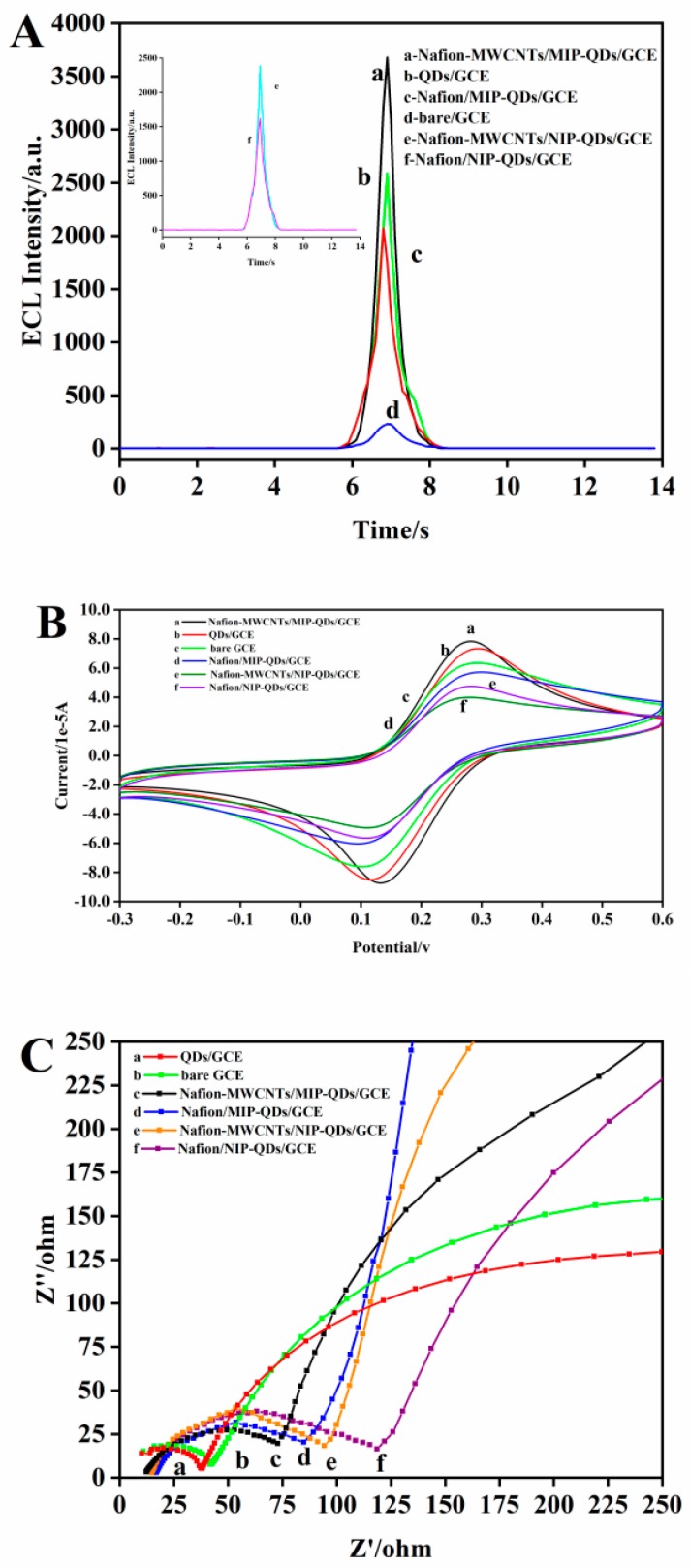
(**A**) ECL curves, (**B**) CVs and (**C**) EIS curves of MIP-QDs/GCE, NIP-QDs/GCE, QDs/GCE, bare GCE. The impedance spectra of frequency range 100 mHz–100 kHz were recorded under an alternating current (AC) amplitude of 10 mV.

The modification of the MIP-QDs on the surface of GCE and the ECL mechanisms of the developed MIECL sensor was explained via the ECL spectra, CV, and EIS. As shown in Figure 3, after the bare GCE was modified with QDs, the ECL intensities (Figure 3A) and reduction–oxidation peak currents (Figure 3B) increased, whereas the electron transfer resistance (Figure 3C) notably decreased. EIS was carried out to characterize the typical impedance spectra. As you can see from Figure 3C, the semicircle diameter in the impedance spectrum equals to the electron transfer resistance, which controls the electron transfer kinetics at the GCE interface. These findings agreed that QDs exhibited good ECL properties and superior conductivity, which could increase their capability for electron transfer [27]. Nevertheless, after the bare GCE was modified with MIP-QDs or NIP-QDs, the ECL intensities and reduction–oxidation peak current evidently decreased compared with those of QDs/GCE, whereas, the electron transfer resistance remarkably increased, thereby indicating that the silica layer on the surface of the MIP-QDs/NIP-QDs possessed poor conductivity and hindered electron transfer and exhibited ECL quenching [28].

As illustrated in Figure 3, although the ECL and reduction–oxidation peak current intensity of MIP-QDs/GCE was lower than that of QDs/GCE, the ECL intensities of MIP-QDs were stronger than those of NIP-QDs. Simultaneously, the MIP-QDs exhibited larger reduction–oxidation peak currents and smaller electron transfer resistance, thereby suggesting that the MIP-QDs possessed higher electrical conductivity compared with NIP-QDs, significantly improving the ECL signals. This property might be ascribed to the numerous selective porous imprinting cavities of the MIP-QDs, which could provide the channels with large surface and pore volume for accelerating electron transfer in the MIECL process [29].

In view of the inhibition of ECL by the silica layer on the MIP-QDs surface, MWCNTs were introduced to the MIP-QDs to further improve the ECL intensity and obtain high sensitivity of the developed MIECL sensor. Figure 3 illustrates that the ECL intensities remarkably improved after the introduction of MWCNTs to the MIP-QDs and NIP-QDs/GCE, whereas the reduction-oxidation peak current (Figure 3B) and electron transfer resistance (Figure 3C) increased and decreased, respectively. These findings suggested that MWCNTs could amplify the ECL signal due to their excellent electrical conductivity and good electrochemical activity, thereby accelerating the electron transfer rate [30].

H_2_O_2_ act as a co-reactant and react with individual reduced nanocrystal material or assembly of reduced nanocrystal materials to produce ECL emissions [31]. Furthermore, according to the spectra patterns of the ECL, CV, and EIS of the QDs, MIP-QDs and NIP-QDs observed in this study, the possible ECL mechanisms could be described as below. First, the ground state QDs were electrochemically reduced to negatively charged QDs^n^*^−^; second, the QDs radicals directly reacted with H_2_O_2_ to become excited QDs*; finally, the light emitted by the QDs* returned to the ground state. The speculated ECL mechanism is presented as follows:

QDs + ne → QDs^n^*^−^ (1)

QDs^n^*^−^ + nH_2_O_2_ → nOH- + QDs* (2)

QDs* → QDs + hv(3)

**Figure 4:**
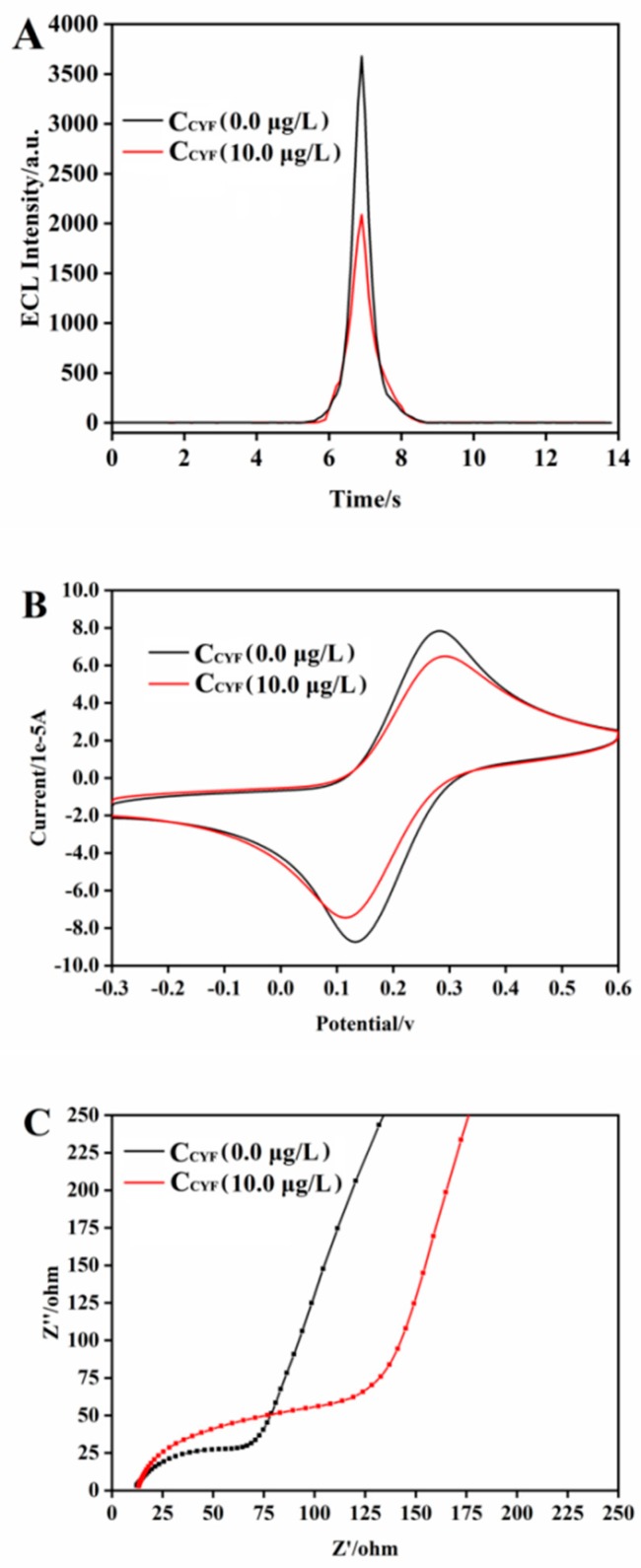
(**A**) ECL curves, (**B**) Cyclic voltammograms and (**C**) EIS curves of Nafion-MWCNTs/MIP-QDs/GCE, before and after adding CYF. CYF concentration (C_CYF_): 10.0 μg/L.

Furthermore, as shown in Figure 4, when the MWCNT-Nafion/MIP-QDs/GCE was incubated in 10.0 µg/L CYF solution, the ECL intensities distinctly decreased. The reduction–oxidation peak current (Figure 4B) also decreased, accompanied by the increased electron transfer resistance (Figure 4C). The possible ECL quenching mechanisms of the developed MIECL sensor based on the MIP-QDs are described as follows. When the MIP-QDs/GCE was incubated with CYF, the specific binding of CYF to the imprinted cavities, which were complementary in size, shape, and functional groups to CYF onto the MIP-QDs, blocked the electron transport channel between QDs/H_2_O_2_ and the GCE, thus causing the specific ECL intensity quenching [32].

### 3.3. Optimization of the MIECL Sensor Conditions

**Figure 5:**
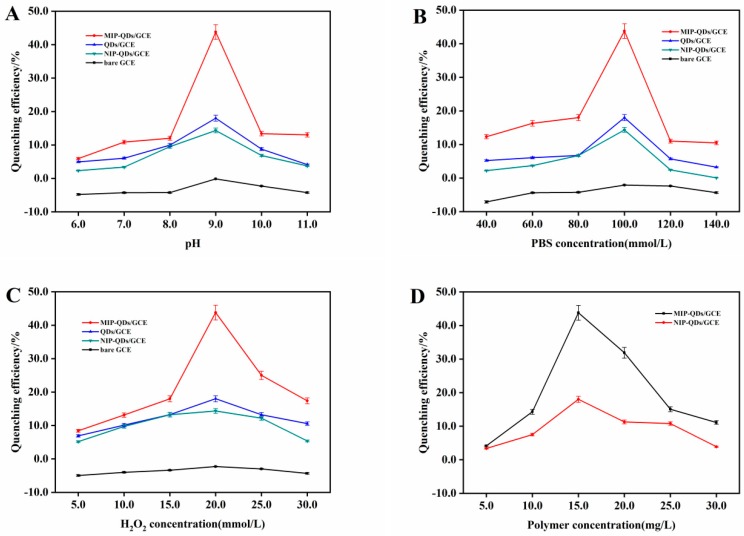
Effects of experimental conditions on the quenching efficiency of MIP-QDs/GCE, NIP- QDs/GCE, bare GCE and QDs/GCE: (**A**) The PBS pH in the ECL cell; (**B**) The concentration of PBS; (**C**) The concentration of H_2_O_2_; (**D**) The concentration of polymers.

The effects of parameters, such as pH and the concentration of the PBS, H_2_O_2_, and MIP-QDs, on the efficiency of ECL quenching, were assessed. As shown in Figure 5A, the maximum ECL quenching efficiency (45.0%) of the developed MIECL sensor was achieved at pH 9.0 because the negatively charged substances could promote electron transfer [33]. When the pH was greater than 9.0, excess anions were loaded on the electrode surface, significantly inhibiting the negative charge of the CdSe-QDs and causing a rapid reduction in quenching efficiency of the MIECL sensor [34].

The effect of PBS concentration on the quenching efficiency of the MIECL sensor was also investigated. As shown in Figure 5B, the quenching efficiency of the MIP-QDs/GCE increased continuously from 40.0 mmol/L to 100.0 mmol/L. When the PBS concentration exceeded 100.0 mmol/L, the quenching efficiency decreased because the ion concentration of the PBS solution effectively promoted the formation of QDs* and enhanced the quenching efficiency. When the concentration of PBS further increased, additional ions aggregated in the polymer cavity, thus hindering the electron transfer on the electrode surface and ultimately resulting in poor quenching efficiency [35].

As the co-reactant, H_2_O_2_ was the most important factor of the developed MIECL sensor in producing ECL signals. As shown in Figure 5C, the quenching efficiency increased with the increase of H_2_O_2_ concentration, reaching the maximum at 20.0 mmol/L. This result was better than obtained with the NIP-QDs/GCE. Meanwhile, excessive H_2_O_2_ easily reacted with the negatively charged cathode, resulting in QDs receiving few electrons, which in turn, inhibited the formation of excited-state QDs* and led to decreased quenching efficiency [36]. Therefore, 20.0 mmol/L was selected as the optimum concentration of co-reactant H_2_O_2_.

As an identification element, the amounts of the MIP-QDs influenced the stability, selectivity, and durability of the working electrode-modified thin layer [37]. As presented in Figure 5D, as the concentration increased from 5.0 mg/L to 15.0 mg/L, the quenching efficiency of the developed MIECL sensor continually increased until 45.0%, which was better than that of MWCNTs-Nafion/NIP-QDs/GCE. Such an increase in quenching efficiency may be caused by the good conductivity of the CdSe-QDs, which remarkably enhanced the mobility of system ions on the surface of the MIP-QDs/GCE, resulting in high quenching efficiency of the developed MIECL sensor for target molecules [38]. However, when the concentration of the MIP-QDs exceeded 15.0 mg/L, the quenching efficiency of the developed MIECL sensor decreased gradually. These results suggested that the electron transfer resistance conferred by the silica layers upon the surface of the MIP-QDs played a dominant role when excess amounts of MIP-QDs were coated onto the GCE, eventually decreasing quenching efficiency. Hence, 15.0 mg/mL MIP-QDs were selected in the fabrication the MIECL sensor.

We observed that the MIP-QDs/GCE (45.0%) exhibited stronger quenching efficiency for CYF than NIP-QDs/GCE (13.0%) within the parameters and scopes of this study. This result further proved that the imprinted silica layers were successfully coated onto the surface of the QDs. Moreover, the specific recognition of imprinting cavities on the surface of the MIP-QDs toward CYF played an important role in the ECL quenching of the developed MIECL sensor.

### 3.4. Selectivity Evaluation of the Developed MICEL Sensor

**Figure 6:**
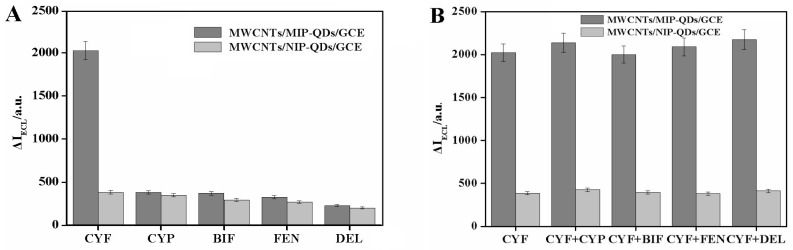
(**A**) Selectivity of the MIECL sensor to CYF and its structural analogs (CYP, BIF, FEN, and DEL) at the same concentration; (**B**) Competitive tests of the MIECL sensor to CYF and its analogues (CYP, BIF, FEN, DEL,). Two-fold concentrations of these interferents were added to 10.0 μg/L CYF.

**Figure 7:**
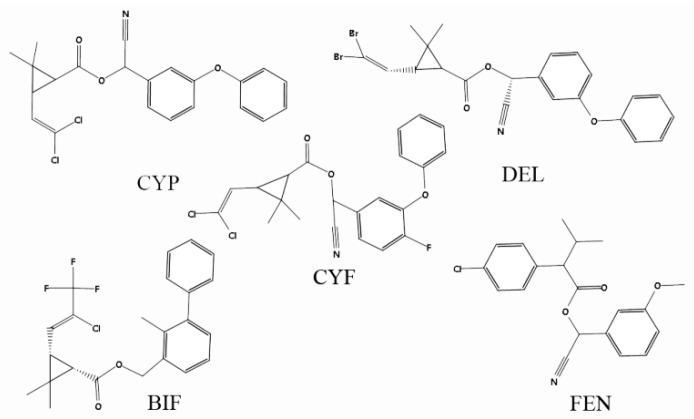
The Chemical structure of CYF and its structural analog.

MIPs possess excellent specificity and selectivity, and template molecules can specifically bind to recognition sites onto the surface of MIPs. Under optimal conditions, selective experiments were carried out to evaluate the recognition capability of the proposed MIECL sensor. Figure 6A showed that the developed MIECL sensor exhibited stronger ECL quenching than NIP-QDs for CYF. The strongest ECL quenching of CYF to the MIP-QDs/GCE was obtained compared with those of its structural analogs, such as (a) BIF, (b) DEL, (c) CYP, and (d) FEN (Figure 7). These results indicated that the specific imprinting recognition sites, with complementary spatial structures and binding sites for CYF, were anchored onto the surface of the MIP-QDs. Meanwhile, the similar low level ECL quenching capability of the NIP-QDs/GCE to CYF and its analogs were detected, thus indicating that no specific recognition coating was fabricated onto the surface of the NIP-QDs/GCE. Furthermore, the selectivity of the MIP-QDs/GCE was confirmed by competitive ECL quenching experiments. As shown in Figure 6B, when the two-fold concentrations of its analogs were mixed to the 10.0 μg/L CYF, there was no significant change in the ECL intensity of the MIP-QDs/GCE, indicating that the MIECL sensor exhibited high selectivity in CYF determination. These results demonstrated that the developed MIP-QDs exhibited excellent selectivity and ECL quenching capability to CYF; furthermore, the specific binding of imprinting cavities to CYF is the major dominating force in the ECL quenching response. 

### 3.5. Application of the MIECL Sensor in the Fish and Seawater Samples 

The practical application of the developed MIECL sensor based on the Nafion-MWCNTs/MIP-QDs/GCE was validated in terms of linearity, sensitivity, stability, reproducibility, accuracy, and precision under optimal conditions.

**Figure 8:**
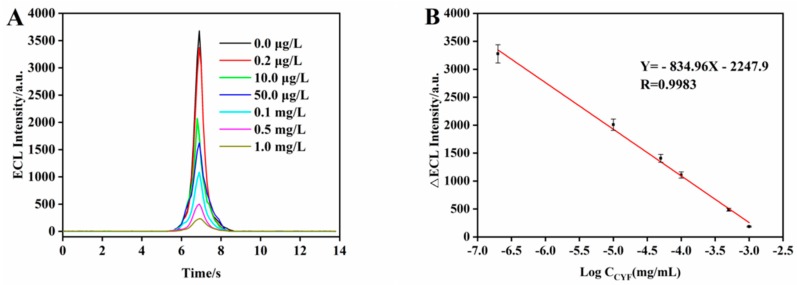
(**A**) ECL responses of the proposed MIECL sensor in the presence of various CYF concentrations (0.2 μg/L, 10.0 μg/L, 50.0 μg/L, 0.1 mg/L, 0.5 mg/L, and 1.0 mg/L); (**B**) calibration curves of the MIECL sensor for CYF determination.

**Figure 9:**
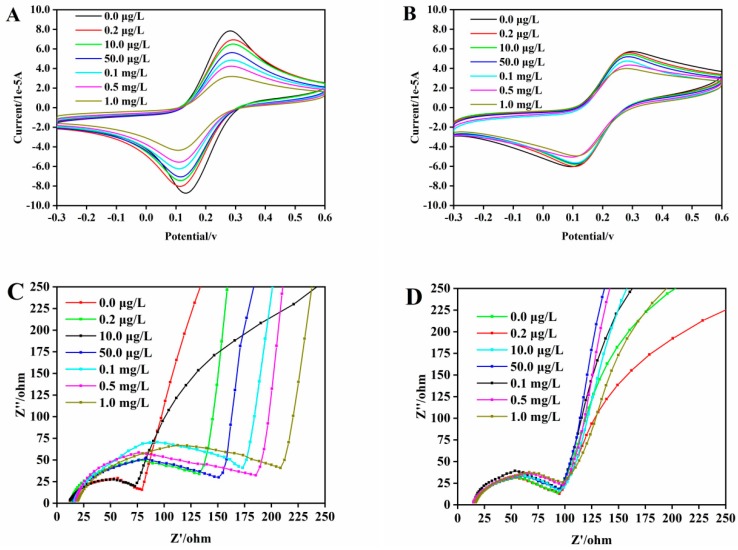
(**A**) CVs of MIP-QDs/GCE; (**B**) NIP-QDs/GCE; (**C**) EIS curves of MIP-QDs/GCE; (**D**)NIP-QDs/GCE responses of the proposed MIECL sensor in the presence of various CYF concentrations (0.2 μg/L, 10.0 μg/L, 50.0 μg/L, 0.1 mg/L, 0.5 mg/L, 1.0 mg/L) in 5.0 mmol/L [Fe(CN)_6_]^3−/4−^ containing 0.1 mol/L KCL solution.

First, we investigated the linear relationship between the CYF concentration and ECL response. As observed in Figure 8A, the ECL intensity decreased gradually as the CYF concentration increased. A good linear relationship between the ECL intensity and the logarithm of CYF concentration was obtained within the CYF concentrations ranging from 0.2 µg/L to 1.0 × 10^3^ μg/L with a determination coefficient (R^2^) of 0.9983 (n = 6, Figure 8B). The calibration curve was described as follows: I = −834.96 logCCYF −2247.9. The calculated limit of detection (LOD) was 0.05 μg/L (S/N = 3). Meanwhile, as shown in Figure 9, the reduction–oxidation peak current of the MIP-QDs/GCE was sequentially decreased in the range of 0.2 μg/L to 1.0 × 10^3^ μg/L. Correspondingly, the electron transfer resistance of MIP-QDs sequentially increased, further demonstrating that the decreased ECL strength of the developed MIECL sensor was ascribed to the blockage electron transfer channel on the surface of MIP-QDs after CYF rebinding [37,39].

**Figure 10:**
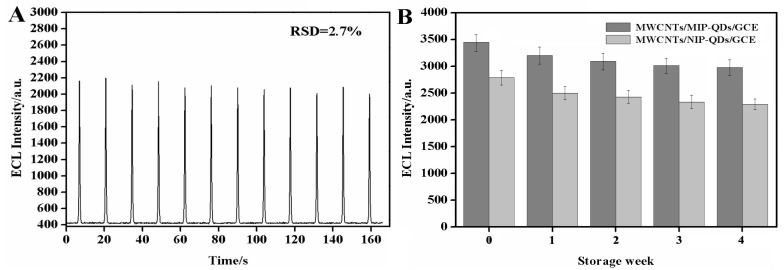
(**A**) ECL stability of the MIECL sensor with 10.0 μg/L CYF in PBS (100.0 mmol/L, pH 9.0) containing 20.0 mmol/L H_2_O_2_; (**B**) Storing stability of the sensor for 0–4 weeks.

Stability and repeatability were considered non-negligible features for evaluating the performance of the MIECL sensor. Figure 10A showed the ECL strength of the MIECL sensor under 12 cycles of continuous cyclic potential scanning and the 2.7% relative standard deviation (RSD) of the ECL signal (10.0 μg/L of CYF), indicating excellent stability of the developed MIECL sensor. At the same time, long-term storage stability was evaluated by monitoring the ECL strength of the developed MIECL sensor. As illustrated in Figure 10B, the ECL strength of the developed sensor was maintained at 85.3% of the original strength after 2 weeks of storage in a refrigerator at 4 °C, thus indicating the satisfactory reproducibility and good stability of the developed MIECL sensor. The above results demonstrated the excellent application potential of the developed MIECL sensor in field detection. 

The developed MIECL sensor was applied to the determination of different CYF concentrations in fish and seawater samples. As summarized in Table 1, the recoveries of CYF in fish samples at three spiked concentrations (10.0, 20.0, and 50.0 µg/kg) ranged from 93.2% to 98.6% with RSDs below 3.6%. The LOD, which was defined as the CYF concentration that could quench three times the standard deviation of the blank sample ECL signal, was 0.22 μg/kg. The recoveries obtained from seawater samples ranged from 86.0% to 98.4%, with RSDs below 4.1%, and LOD was 0.11 μg/L, at concentrations of 1.0, 2.0, and 5.0 µg/L. These results indicated the excellent practical application of the MIECL sensor based on the MIP-QDs for accurate CYF determination.

Finally, the developed MIECL sensor in this study demonstrated excellent performance with better sensitivity, wide linear range, accuracy, precision, and reproducibility compared to other detection methods (Table 2).

## 4. Conclusions

The MIECL sensor based on the MIP-QDs was fabricated for the first time for CYF determination in seawater and fish samples. The MIECL mechanisms based on the MIP-QDs with H_2_O_2_ as co-reactant were explained, and its introduction into the Nafion ion exchange membrane significantly enhanced the ECL signal of the MIECL sensor, which is due to the excellent electrical conductivity of MWCNTs. Apart from its successful application to CYF determination in fish and seawater samples, the developed MIECL sensor showed good sensitivity, selectivity, stability, and linearity. The excellent performance of the MIECL sensor provides a potential strategy for the sensitive and selective determination of CYF residues in aquaculture biology and environment samples.

## Figures and Tables

**Table 1:** Results of ECL measurements of CYF content in real samples using the proposed MIECLS.

Sample	Added (μg/kg, L)	Found (μg/kg, L)	RSD (%) (n = 3)	Recovery (%) (n = 3)
	10.0	9.32	3.6	93.2
Fish	20.0	19.72	3.2	98.6
	50.0	47.87	2.2	95.7
	1.0	0.86	1.5	86.0
Seawater	2.0	1.76	2.5	88.0
	5.0	4.92	4.1	98.4

**Table 2:** Comparison of this method with other methods reported in the literatures.

Detection Method	Target Object	Linear Range (µg/kg, L)	LOD (µg/kg, L)	Recovery (%)	Reference
SiO_2_-MPTMS @FMIPs	Beta-Cyfluthrin	4.6–36.0	4.55	103.8–111.8	[40]
Inorganic-organic MIP-coated QDs	Cyphenothrin	37.5–3.0 × 10^4^	3.40	88.5–97.1	[41]
MIPs-OVDAC/CdTe QDs	λ-Cyhalothrin	45.0–7.2 × 10^3^	13.5	97.3–105.5	[42]
MIP on FeSe QDs	Cyfluthrin	10.0–2.0 × 10^2^	1.00	88.0–113.9	[24]
Dy(III)@SiO2@MIPs	λ-cyhalothrin	4.5 × 10^3^–4.5 × 10^5^	719.7	102.7–107.9	[43]
Nafion-MWCNTs/MIP-CdSe QDs	Cyfluthrin	0.2–1.0 × 10^3^	0.05	86.0–98.6	This work
